# Dental Coverage Through Medicaid Managed Care vs Fee-for-Service

**DOI:** 10.1001/jamahealthforum.2025.6958

**Published:** 2026-02-27

**Authors:** Hawazin W. Elani, Ningsheng Zhao, Jacob Wallace, Benjamin D. Sommers

**Affiliations:** 1 Department of Oral Health Policy and Epidemiology, Harvard School of Dental Medicine, Harvard University, Boston, Massachusetts; 2 Department of Health Policy and Management, T.H. Chan School of Public Health, Harvard University, Boston, Massachusetts; 3 School of Public Health, Yale University, New Haven, Connecticut; 4 Department of Medicine, Brigham and Women’s Hospital, Harvard Medical School, Boston, Massachusetts

## Abstract

**Importance:**

Adult dental coverage under Medicaid varies widely across states and delivery systems. Most adult beneficiaries are now enrolled in managed care, yet little is known about how these benefits are structured and implemented within Medicaid managed care organizations (MCOs).

**Objective:**

To examine trends in the generosity, delivery models, and enrollment patterns of adult dental benefits delivered through Medicaid MCOs and assess coverage alignment with Medicaid fee-for-service (FFS) coverage.

**Design, Setting, and Participants:**

This cross-sectional analysis included adult Medicaid beneficiaries aged 19 years or older who had state Medicaid MCO or FFS coverage across all 50 states and Washington, DC. This analysis used a novel linked national dataset combining multiple Centers for Medicare & Medicaid Services administrative sources from 2016 to 2022. The analysis was conducted from April 8, 2025, to October 8, 2025.

**Exposures:**

State-level Medicaid MCO or FFS program with adult comprehensive dental coverage, emergency-only dental coverage, or no dental coverage.

**Main Outcomes and Measures:**

The generosity of dental benefits; the degree of alignment in benefit generosity between Medicaid MCO and Medicaid FFS programs; and enrollment of adults in dental MCO or FFS programs by benefit (coverage) type.

**Results:**

The number of states offering comprehensive adult dental benefits through Medicaid MCOs increased from 33 (64.7%) in 2016 to 36 (70.6%) in 2022. Carve-out models, typically using stand-alone prepaid health plans for dental coverage, increased from 4 states (7.8%) in 2016 to 8 states (15.7%) in 2022. In 2016, 51.0% of states had mismatched benefit levels between MCO and FFS programs compared with 35.3% in 2022. The number of MCO enrollees with comprehensive dental coverage increased from 14.2 million (58.7%) in 2016 to 25.3 million (59.8%) in 2022, reflecting substantial managed care penetration.

**Conclusions and Relevance:**

This study found that although the generosity and scope of MCO dental benefits expanded over time, alignment with FFS programs remained inconsistent. As new legislation cuts federal funding to states, understanding how dental benefits are designed and delivered is critical to inform future coverage decisions and ensure equitable access.

## Key Points

**Question:**

How do states structure and deliver adult dental benefits through Medicaid managed care organizations (MCOs), and how do these benefits align with Medicaid fee-for-service (FFS) coverage?

**Findings:**

In this cross-sectional analysis of Medicaid programs in all 50 states and Washington, DC, the share of states offering comprehensive dental benefits through MCOs increased from 64.7% in 2016 to 70.6% in 2022. However, benefit generosity between MCO and FFS programs remained misaligned across states despite improvements over time.

**Meaning:**

State approaches to Medicaid managed care dental coverage remain heterogeneous; strengthening alignment across delivery systems can help promote equitable access to adult dental care.

## Teaser

This cross-sectional analysis examines trends in the generosity, delivery models, and enrollment patterns of adult dental benefits delivered through Medicaid managed care compared with Medicaid fee-for-service programs.

## Introduction

Adult dental coverage under Medicaid remains one of the most inconsistently provided benefits in public insurance programs.^1^ Adult dental benefits are not federally mandated, giving states broad discretion over whether, and to what extent, these services are covered. As a result, coverage varies widely across states, as well as within states over time. Some states offer comprehensive adult dental benefits, whereas others only provide emergency services or do not provide coverage at all. Even among states with comprehensive dental coverage, the scope ranges from preventive and basic restorative services to more extensive procedures such as root canals.^2^

Although prior research on Medicaid dental policy has largely focused on fee-for-service (FFS) programs, 75% of adult Medicaid beneficiaries now receive care through Medicaid managed care.^3^ In this model, states contract with managed care organizations (MCOs), which are private entities responsible for delivering Medicaid benefits, including dental services in many states. These dental benefits may be administered directly by MCOs as part of a comprehensive contract (carve in) or through separate arrangements with dental-specific plans (carve out).^3^ Despite the increasing role of managed care in delivering adult dental services, little is known about how these benefits are structured across states. Although national studies have examined trends in managed care enrollment and medical service delivery, few have focused on the generosity of dental benefits, delivery model design, or how dental benefits in MCOs align with FFS programs.^4,5^ Research on Medicaid managed care has focused primarily on dental benefits for children,^6^ leaving gaps in the understanding and implementation of dental benefits for adults.

Understanding how states deliver adult dental benefits through managed care is important for several reasons. First, inadequate dental coverage is associated with preventable pain, avoidable emergency department visits, and higher downstream medical costs.^7,8,9^ Second, the differences in benefit design between the MCO and FFS programs can create confusion for beneficiaries and disrupt continuity of care, particularly for individuals who transition between delivery systems (MCO or FFS). Third, dental benefits in MCOs are often difficult to monitor because coverage details vary and are not consistently reported. Fourth, dentist networks may differ between MCO and FFS programs, which can affect access to dental care and dentist participation. Fifth, state decisions about scope and delivery model structure for dental benefits may shape enrollment patterns and access. These issues have gained renewed urgency amid new federal legislation to reduce Medicaid spending, including the budget reconciliation act of 2025 (HR 1; Public Law 119-21). The Congressional Budget Office estimates this law^10^ will reduce federal Medicaid spending by $911 billion over a decade and limit current revenue sources for the states’ share of program costs. In response to fiscal pressures, some states may consider expanding the use of managed care or restructuring dental benefits to contain costs, making it critical to understand how these programs are currently designed and administered.

To address these gaps, we constructed a dataset capturing adult dental benefits delivered through Medicaid MCO and FFS programs in all 50 states and Washington, DC, from 2016 to 2022. We examined trends in dental benefit delivery, including changes in program offerings, generosity, and delivery model structure. We also examined differences in dental benefit generosity between MCO and FFS programs by year in each state. Next, we analyzed how adult Medicaid enrollment was distributed across dental benefit types and delivery models (MCO or FFS) by year in each state.

## Methods

This study was determined not to be human participant research by the institutional review board of the Harvard Faculty of Medicine, which waived informed consent. The Strengthening the Reporting of Observational Studies in Epidemiology (STROBE) reporting guideline was used to conduct this cross-sectional study. The analysis was conducted from April 8, 2025, to October 8, 2025.

### Data Sources

#### Identifying Eligibility

We used a Centers for Medicare & Medicaid Services (CMS) dataset^11^ to identify Medicaid MCO programs that included dental benefits for adults. We included data from 2016 and 2022, which were the first and most recent years for which these data were available (eMethods 1 in Supplement 1). For each MCO program, we extracted indicators of dental benefit generosity for each year. Due to limited enrollment and distinct eligibility rules, we excluded Programs of All-Inclusive Care for the Elderly. We determined whether each MCO program served adults by using CMS codes that defined the eligibility groups and supplemented this search with manual review (eMethods 2-3 in Supplement 1).

#### Estimating Enrollment

##### MCO Programs

Because the CMS does not directly report adult dental enrollment, we used a multistep process to estimate enrollment among adults receiving dental benefits through managed care plans. We merged 2 CMS datasets; one dataset includes total enrollment at the plan level^12^ and the other dataset provides dental benefit information at the program level.^11^ Because dental benefits are not reported at the plan level, we linked plans to their corresponding programs using exact name matching, fuzzy string matching, and cosine similarity over the TF-IDF vectorization^13^ (eMethods 4 in Supplement 1).

We then calculated total enrollment in dental MCO programs (eMethods 5 in Supplement 1). To estimate adult enrollment, we subtracted child enrollment from the total using data from a CMS report.^14^ Data exclusions and imputations are described in eMethods 1 in Supplement 1.

We also used data from KFF^15^ to obtain supplemental enrollment distributions by eligibility groups at the state level.

##### FFS Programs

To estimate enrollment in Medicaid FFS dental programs, we used data from the CMS Transformed Medicaid Statistical Information System monthly enrollment files by eligibility group; the enrollment files provide total adult Medicaid enrollment by state.^16^ State- and year-specific indicators of adult dental benefit generosity also were used and were derived from our Medicaid FFS state dental benefit dataset^2^ to estimate the share of each state’s adult Medicaid FFS population with dental coverage (eMethods 5 in Supplement 1).

### Study Population

The study population included Medicaid beneficiaries aged 19 years or older. Depending on the year, between 9 and 14 states did not provide adult dental coverage in managed care programs and neither Alaska nor Connecticut implemented managed care programs at any point (eTable in Supplement 1).

### Statistical Analysis

To assess trends in adult dental benefit generosity in managed care across states and over time, we created a categorical measure of MCO dental benefit generosity for each state and year based on CMS definitions for adult Medicaid coverage.^17^ Benefits were classified as no coverage (no adult dental services), emergency-only dental coverage (limited to treatment of acute pain, infection, or trauma), limited dental coverage (<100 diagnostic, preventive, and minor restorative services with an annual cost cap of ≤$1000), and extensive dental coverage (>100 diagnostic, preventive, and restorative services with an annual cost cap of ≥$1000). Because the CMS dataset^11^ only reports MCO dental benefits as emergency-only or comprehensive coverage, we combined the limited and extensive categories into a single comprehensive group. Accordingly, we classified adults as having comprehensive dental benefit coverage, emergency-only coverage, or no dental coverage.

We described annual trends from 2016 to 2022 and summarized the distribution of each benefit type by year. We then linked benefit types to enrollments to examine changes in MCO enrollment by benefit type. We also examined benefit generosity by program delivery characteristics, including mandatory vs voluntary enrollment, statewide vs regional implementation, and carve-in vs carve-out models.

To evaluate misalignment between dental benefits provided by MCO and FFS programs, we compared benefit generosity across delivery models within each state and by year using a 3-level scale (comprehensive coverage, emergency-only coverage, or no dental benefits). We constructed an alignment indicator to classify each state by year as aligned (equivalent in both systems including the category of no coverage in a MCO vs a FFS program), more generous in a MCO program, or more generous in a FFS program. We then calculated the proportion of states in each category for every year from 2016 to 2022.

All analyses were conducted using Python version 3.13.

## Results

Between 2016 and 2022, the generosity of adult dental benefits offered through MCO programs varied across states and over time. Statewide mandatory enrollment was the most common and increased over time. In 2022, 34 states (66.7%) operated MCO programs with mandatory, statewide dental benefit enrollment compared with 31 states (60.8%) in 2016 (Table and eFigures 1-2 in Supplement 1).

**Table.** Characteristics of Medicaid Managed Care Dental Coverage, Delivery Models, and Program Types^a^

Characteristic	Year						
	2016	2017	2018	2019	2020	2021	2022
Managed care delivery model structure, No. of states (%)^b^							
Mandatory statewide	31 (60.8)	31 (60.8)	35 (68.6)	35 (68.6)	31 (60.8)	34 (66.7)	34 (66.7)
Voluntary statewide	8 (15.7)	6 (11.8)	4 (7.8)	4 (7.8)	4 (7.8)	4 (7.8)	4 (7.8)
Mandatory partially	5 (9.8)	3 (5.9)	4 (7.8)	5 (9.8)	5 (9.8)	4 (7.8)	4 (7.8)
Voluntary partially	2 (3.9)	3 (5.9)	4 (7.8)	3 (5.9)	4 (7.8)	2 (3.9)	3 (5.9)
Dental benefit structure, No. of states (%)^b^							
Carve-in model	33 (64.7)	30 (58.8)	29 (56.9)	30 (58.8)	28 (54.9)	30 (58.8)	30 (58.8)
Carve-out model	4 (7.8)	5 (9.8)	8 (15.7)	8 (15.7)	8 (15.7)	8 (15.7)	8 (15.7)
No benefit	14 (27.5)	16 (31.4)	14 (27.5)	13 (25.5)	15 (29.4)	13 (25.5)	13 (25.5)
Medicaid managed care program types, No. of programs (%)^c^							
Behavioral health	0	0	0	0	0	1 (1.8)	1 (1.7)
Comprehensive MCO with or without MLTSS	47 (81.0)	45 (86.5)	45 (78.9)	44 (80.0)	41 (80.4)	43 (76.8)	44 (75.9)
Dental PAHP	5 (8.6)	6 (11.5)	9 (15.8)	9 (16.4)	9 (17.4)	10 (17.9)	9 (15.5)
MLTSS only	0	0	0	0	0	1 (1.8)	1 (1.7)
PCCM	6 (10.3)	1 (1.9)	3 (5.3)	2 (3.6)	1 (2.0)	1 (1.8)	3 (5.2)

Abbreviations: MCO, managed care organization; MLTSS, managed long-term services and supports; PAHP, prepaid ambulatory health plan; PCCM, primary care case management.

^a^ The data for this analysis were provided by the Centers for Medicare & Medicaid Services Medicaid Datasets and state Medicaid dental coverage for 2016-2022.

^b^ The denominator for the percentages was 51 for the 50 states and the District of Columbia.

^c^ Counts reflect the number of distinct Medicaid managed care programs operating nationally each year; states may run multiple program types concurrently.

Most states used carve-in models, but their use declined slightly (33 states [64.7%] in 2016 to 30 states [58.8%] in 2022). Meanwhile, carve-out models that were typically administered through stand-alone dental prepaid ambulatory health plans (PAHPs) increased from 4 states (7.8%) in 2016 to 8 states (15.7%) in 2022.

**Figure 1.**
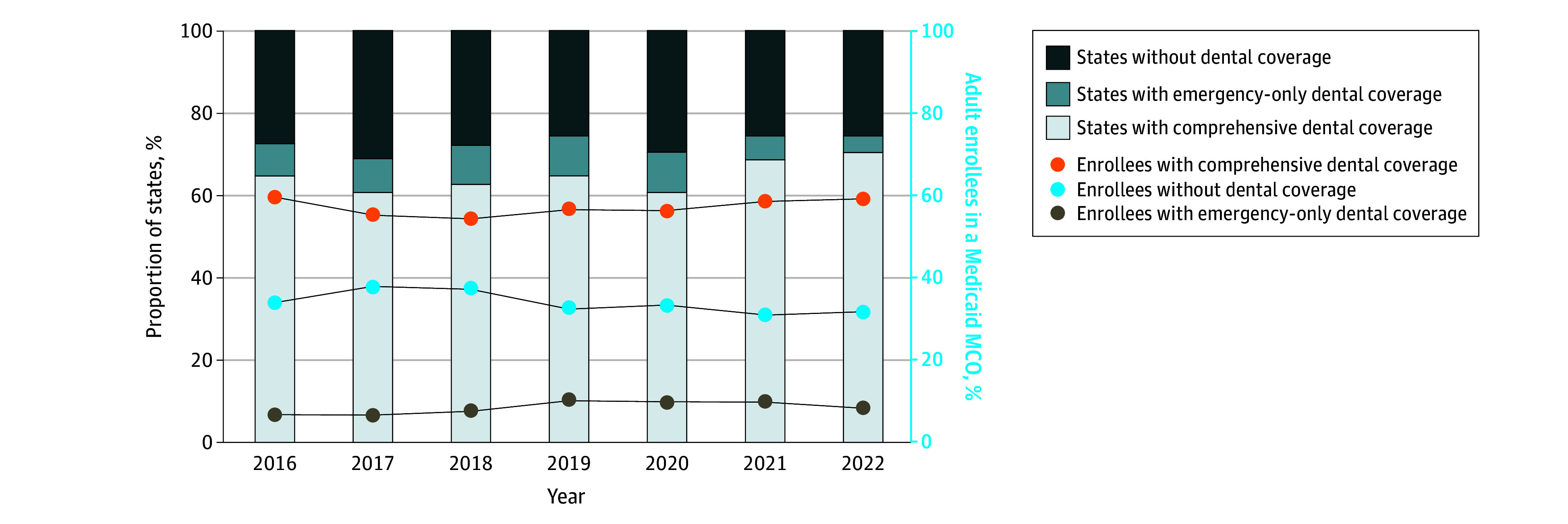
Component Bar Graphs and Line Graphs Displaying Trends in Adult Dental Coverage in Medicaid Managed Care Organizations (MCOs) by State and Enrollee The data for this analysis were provided by the Centers for Medicare & Medicaid Services Medicaid Datasets and state Medicaid dental coverage for 2016-2022. The bar segments represent the proportion of states with MCOs providing no adult dental coverage, MCOs providing emergency-only coverage, or MCOs providing comprehensive coverage. The lines represent the percentage of adult enrollees in MCOs with comprehensive dental benefits, no dental benefits, or emergency-only dental benefits. Comprehensive coverage includes preventive and restorative services such as cleanings, fillings, and crowns. Emergency-only coverage provides limited, medically necessary services like extractions for pain or infection.

The number of states offering comprehensive dental coverage through MCOs increased from 33 (64.7%) in 2016 to 36 (70.6%) in 2022 (Figure 1). States providing no adult dental coverage through MCOs (meaning benefits were available only through FFS programs) declined from 12 states (23.5%) in 2016 to 11 states (21.6%) in 2022; 2 states did not offer adult dental coverage in either delivery system in 2022.

Comprehensive MCOs remained the dominant program type delivering adult dental benefits. In 2022, 75.9% of programs were MCOs with comprehensive dental benefits (with or without managed long-term services and supports). Other program types accounted for a small share of total dental program offerings (eFigures 3-4 in Supplement 1).

### Policy Misalignment Between MCOs and FFS Programs

**Figure 2.**
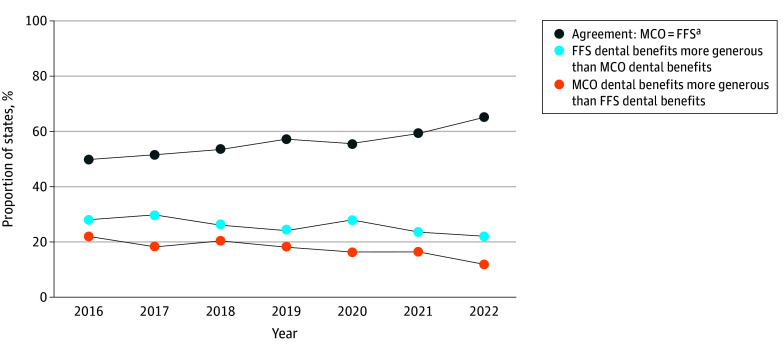
Line Graphs Displaying Trends in Agreement Between Medicaid Managed Care Organization (MCO) and Fee-for-Service (FFS) Adult Dental Benefits The data for this analysis were provided by the Centers for Medicare & Medicaid Services Medicaid Datasets and state Medicaid dental coverage for 2016-2022. ^a^Represents the proportion of states where adult dental benefit levels were the same in both Medicaid MCO and FFS programs and included cases in which no adult dental benefit was offered in either system.

There was frequent misalignment in adult dental benefit generosity between MCO and FFS programs. However, the proportion of states with aligned benefit levels (defined as offering the same level of dental coverage in both MCO and FFS programs) increased from 49.0% in 2016 to 64.7% in 2022 (Figure 2). States offering more generous dental benefits in MCOs decreased from 21.6% in 2016 to 11.8% in 2022. States offering more generous dental benefits in FFS programs decreased from 27.4% in 2016 to 21.5% in 2022.

**Figure 3.**
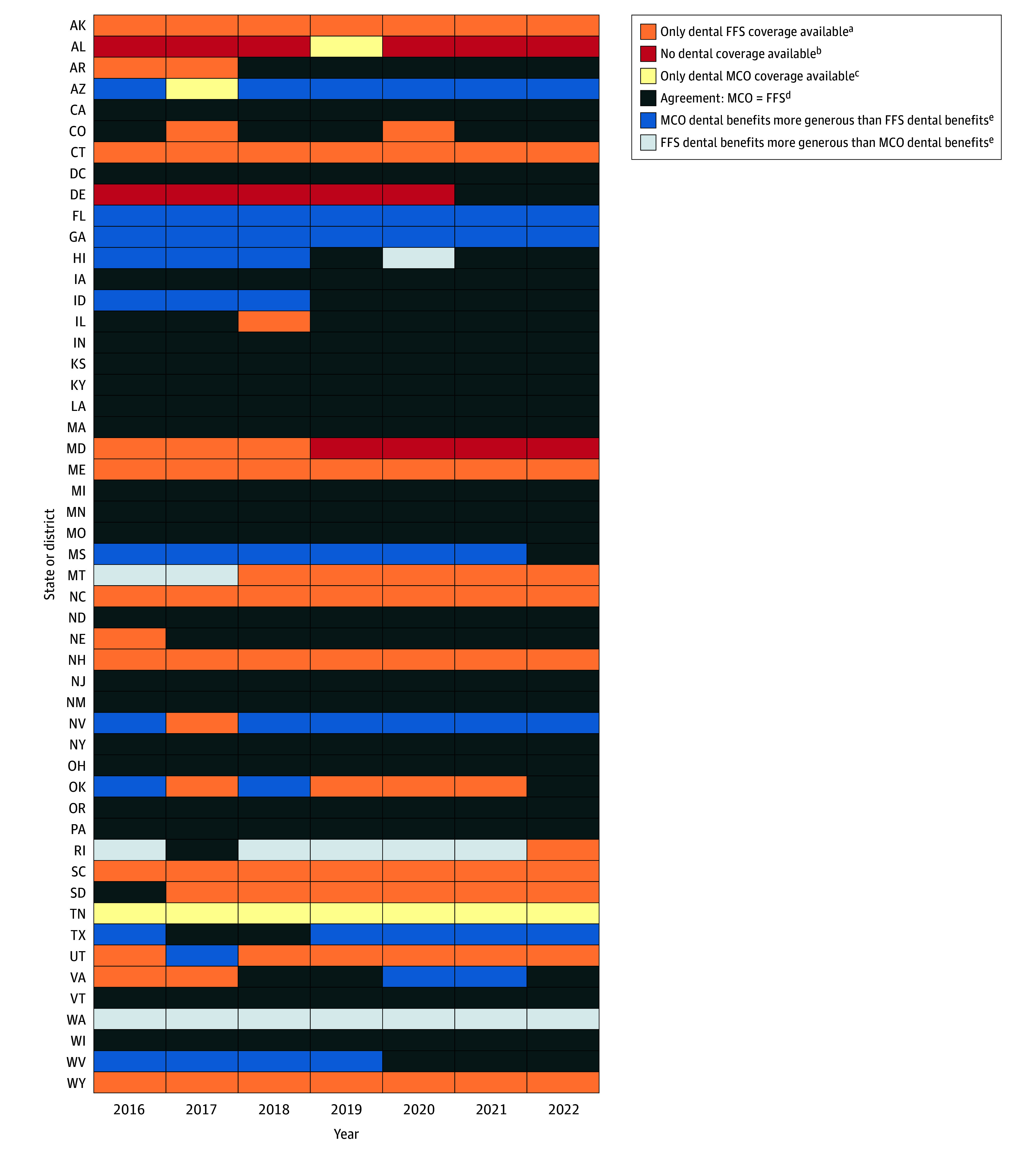
Heat Map Displaying Annual State-Level Differences in Adult Dental Benefit Generosity Between Managed Care Organization (MCO) and Fee-for-Service (FFS) Programs for 2016-2022 The MCO benefit levels are reported annually. The FFS benefit levels were available monthly; therefore, for states with within-year changes, the annual FFS benefit category was based on the benefit in effect for the majority of the year (>6 months). For states with exactly 6 months in each benefit category, we used the benefit in effect during the first half of the year. ^a^States that provided adult dental benefits only through FFS Medicaid. ^b^States without adult dental benefits in either Medicaid delivery model. ^c^States that offered adult dental benefits exclusively through MCOs. ^d^States in which the MCOs and FFS programs provided the same level of dental benefits in a given year. ^e^States in which both delivery models offered adult dental benefits but differed in benefit generosity.

Several states showed persistent divergence. For example, Florida and Georgia consistently provided more generous dental benefits via MCOs than FFS programs, whereas the state of Washington maintained more generous dental benefits in FFS programs throughout the assessment period (Figure 3).

Throughout the study period, among states that offered adult dental benefits, the MCO programs were more likely than the FFS programs to offer more generous dental coverage (eFigures 5-6 in Supplement 1). In 2016, 10 states (19.6%) had MCO programs with more generous dental benefits, whereas only 3 states (5.9%) had FFS programs with more generous benefits. By 2022, 5 states (9.8%) had MCO programs with more generous dental benefits and 1 state (2.0%) had FFS programs with more generous coverage. In 2016, 51.0% of states had mismatched benefit levels between MCO and FFS programs compared with 35.3% in 2022.

### Enrollment Patterns and Policy Generosity

**Figure 4.**
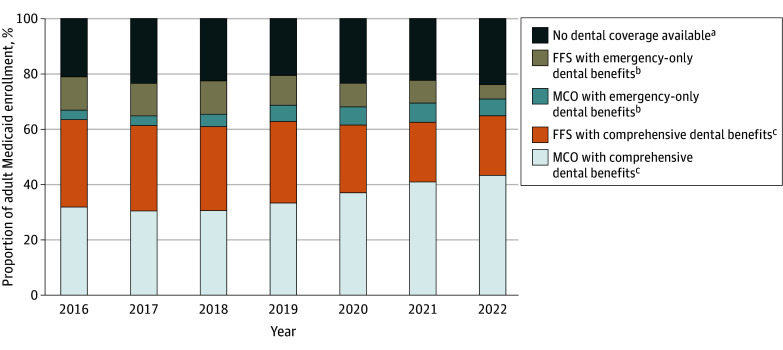
Component Bar Graphs Showing Distribution of Medicaid Adult Enrollment by Type of Dental Coverage and Delivery Model for 2016-2022 FFS indicates fee-for-service; MCO, managed care organization. ^a^Programs that did not offer any dental benefits through either MCO or FFS in a given year. ^b^Provides urgent or medically necessary dental care only. ^c^Includes preventive and restorative services (eg, cleanings, fillings, crowns).

In states offering comprehensive dental benefits through managed care, there were more adults enrolled in dental MCOs (43.4%) in 2022 compared with states offering emergency-only dental coverage (6.0%) (Figure 4). States with mandatory, statewide enrollment had the highest enrollment (76.9%), whereas states with partial or voluntary arrangements showed much lower enrollment (eFigure 2 in Supplement 1). The MCO penetration for comprehensive adult dental benefits increased from 32.0% in 2016 to 43.4% in 2022. The share of adults without any dental coverage (MCO or FFS) increased from 21.0% in 2016 to 24.0% in 2022.

The number of MCO enrollees with comprehensive dental coverage increased from 14.2 million (58.7%) in 2016 to 25.3 million (59.8%) in 2022, reflecting substantial managed care penetration. Among enrollees, emergency-only dental coverage increased from 6.8% (1.6 million) in 2016 to 8.3% (3.5 million) in 2022 (Figure 1).

Enrollment also differed by delivery program (comprehensive MCOs, dental PAHPs, and other programs). In 2022, 53.5% of enrollees in MCOs received dental benefits through comprehensive MCOs (eFigure 7 in Supplement 1). Enrollment in dental PAHPs increased from 5.2% in 2016 to 11.6% in 2022.

Program offerings and enrollment varied widely across states. New York had the largest managed care system for dental care, enrolling 3.45 million adult Medicaid beneficiaries across 2 MCO programs in 2022 (eFigure 8 in Supplement 1).

## Discussion

We found substantial state-level variation in both the availability and generosity of dental benefits offered through MCOs. Over time, more states adopted comprehensive dental benefits within MCOs, and states offering any managed care dental coverage enrolled a larger share of adult Medicaid beneficiaries in these programs. However, alignment between MCO and FFS dental benefit generosity was inconsistent, contributing to fragmentation in coverage and access.

Variation in delivery system and benefit generosity raise concerns because inconsistent dental coverage can lead to poorer oral health and higher spending. Adults with limited or no dental benefits are more likely to delay care and visit the emergency department for avoidable dental conditions.^7^ In states where adult dental benefits were offered only through MCOs, or only through FFS, beneficiaries’ access may depend not only on where they live but also on how they were enrolled. For example, during the study period, Tennessee was the only state that consistently delivered adult dental coverage exclusively through MCOs, with Arizona (in 2017) and Alabama (in 2019) representing isolated cases. Although this approach may increase coverage availability for MCO enrollees, beneficiaries remaining in FFS programs (often those with limited eligibility or residing in regions without MCO contracts) had to enroll in a dental-only MCO plan to obtain coverage, adding administrative steps and potential barriers to accessing dental care. Understanding how plan design and network capacity shape access in these settings remains an important area for future research.

Alignment across delivery systems is generally preferable because the scope of Medicaid adult dental benefits is a state policy choice, and beneficiaries within the same MCO or FFS program should not receive different benefit packages solely due to delivery system enrollment. Differences in benefit generosity can introduce inequities, complicate communication, and disrupt continuity of coverage when individuals transition between MCO and FFS programs due to eligibility changes, geographic moves, auto-assignment policies, or administrative churn. Misalignment can arise for different reasons and has different implications. In some states, broader MCO benefits may reflect intentional expansion beyond a limited FFS package; in others, discrepancies reflect incremental or uncoordinated policy updates across delivery systems. Documenting alignment, therefore, helps to identify where beneficiaries may face different coverage rules within the same state and helps to clarify how states operationalize adult dental benefits across Medicaid delivery systems.

Persistent misalignment between MCO and FFS benefit generosity can introduce challenges for both beneficiaries and state Medicaid agencies. Similar coordination challenges have been documented for behavioral health, in which states frequently operate both MCO and FFS models that increase administrative complexity and complicate access.^18^ As states continue to implement delivery system changes through waivers or payment reforms, greater coordination across delivery systems will become increasingly important.^3^ In states where beneficiaries can choose between MCO and FFS options, these differences in benefit generosity may influence enrollment decisions, although such choice is often limited to specific regions or eligibility groups.

We also found that program features were associated with higher adult enrollment in dental MCOs. Comprehensive benefits, carve-out arrangements, and mandatory enrollment were both associated with greater MCO enrollment, suggesting that benefit scope and program design may shape coverage distribution. However, it remains unclear whether observed enrollment patterns reflect beneficiary preferences, plan offerings, or administrative design. Although we did not examine service use directly, the positive association between benefit generosity and enrollment suggests that delivery system design may influence coverage and possibly plan use.

States take different approaches to structuring dental benefits through carve-in or carve-out models.^19^ Carve-in arrangements, in which dental benefits are integrated into comprehensive MCO contracts, may streamline administration and integration with other Medicaid services, but dental care can receive limited focus within large medical contracts, complicating network oversight. Carve-out models, typically administered through dental PAHPs, allow states to apply dental-specific contracting requirements, performance incentives, and network adequacy standards.^20^ However, carve-out arrangements add another layer to program administration and may increase coordination challenges between dental and medical services. Evidence from behavioral health comparing Medicaid carve-in and carve-out models is mixed, and performance depends on state contracting design, oversight capacity, and reimbursement structures.^21^

In addition, the increasing use of carve-out models (particularly dental PAHPs) reflects a shift toward specialized approaches to dental benefit delivery.^4,5^ These arrangements may support oversight, dentist participation,^22^ and cost control, but they also introduce administrative complexity and do not necessarily address long-standing concerns about network adequacy in Medicaid dental care.^23^ Importantly, benefit generosity and alignment do not necessarily translate into access. More generous benefits offered through managed care may coexist with limited clinician participation and inadequate networks. As a result, assessing benefit alignment should be complemented by measures of dentist availability, network adequacy, and care use.

States operating multiple MCO and PAHP programs may face challenges in ensuring integration with medical services and maintaining consistent communication with beneficiaries. Given the increasing complexity of Medicaid dental delivery systems, standardized and transparent reporting on benefit generosity, enrollment, and networks will be essential for oversight and policy decisions. State MCO programs are also subject to federal reporting requirements^24^ that establish expectations for network adequacy, wait-time reporting, and transparency in plan performance. As states work to meet these requirements, the fiscal constraints introduced by Public Law 119-21^10^ may affect their capacity to build and sustain the reporting and oversight infrastructure needed to comply with these standards.

This national analysis of adult dental coverage under Medicaid managed care highlights substantial variation in benefit generosity, delivery model structure, and alignment with FFS programs. Strengthening managed care design, oversight, and integration may improve consistency and access. However, recent shifts in the Medicaid landscape may threaten progress if fiscal pressures lead states to reduce or eliminate adult dental benefits. Future research should examine how differences in benefit scope and delivery models affect care use, dentist networks, and oral health outcomes for low-income adults.

### Limitations

This study has several limitations. First, estimates of adult dental MCO enrollment were calculated by combining data from the CMS and KFF rather than using directly reported enrollment counts. Therefore, enrollment estimates for some states and years may be subject to measurement error due to limitations in data quality.

Second, we could not assess dental service use or oral health outcomes due to lack of available data. Third, our analysis covers the period from 2016 to 2022, the most recent years for which there are complete data. Programmatic or policy changes after 2022 are not reflected in our findings.

Fourth, we were unable to exclude Medicaid enrollees aged 65 years or older from our enrollment estimates; however, benefit generosity measures were coded at the program level for state and year and were not based on individual dual eligibility.

## Conclusions

This study found that although the generosity and scope of MCO dental benefits expanded over time, alignment with FFS programs remained inconsistent. As new legislation cuts federal funding to states, understanding how dental benefits are designed and delivered is critical to inform future coverage decisions and ensure equitable access.

## Notes

Supplement 1. **eMethods 1.** Study sample construction and subgroup dentitions **eMethods 2.** Identifying dental MCO program offerings and adult eligibility, 2016–2022 **eMethods 3.** Reclassification of eligibility indicators for Medicaid MCO programs with inconsistent or missing enrollment labels **eMethods 4.** Linking CMS datasets to identify dental MCO enrollment **eMethods 5.** Derivation of adult dental enrollment by delivery system, 2016–2022 **eTable.** States without adult dental managed care organizations, 2016–2022 **eFigure 1.** Trends in state adoption of dental Medicaid managed care programs by delivery model and enrollment mandate, 2016–2022 **eFigure 2.** State level MCO dental benefit coverage areas and enrollment mandate for low-income adults, 2016-2022 **eFigure 3.** Distribution of Medicaid managed care program types offering any adult dental benefits, by service type, 2016–2022 **eFigure 4.** State-level Medicaid MCO dental benefit structure (carved-out vs carved-in), 2016-2022 **eFigure 5.** State-level dental benefit generosity by MCO and FFS, 2016-2022 **eFigure 6.** Trends in alignment of adult dental benefits between Medicaid MCO and FFS programs, 2016–2022 **eFigure 7.** Distribution of dental benefit coverage among adult Medicaid MCO enrollees, by program type, 2016–2022 **eFigure 8.** Number of dental Medicaid managed care program adult enrollment by offerings, by state, 2022
eReferences

Supplement 2.
Data sharing statement
