# Supplementary material for: Dental Coverage Through Medicaid Managed Care vs Fee-for-Service
Source: JAMA Health Forum. 2026 Feb 27;7(2):e256958. doi: 10.1001/jamahealthforum.2025.6958 (PMC12949447; doi:10.1001/jamahealthforum.2025.6958)
Supplement: Supplement 1. — eMethods 1. Study sample construction and subgroup dentitions eMethods 2. Identifying dental MCO program offerings and adult eligibility, 2016–2022 eMethods 3. Reclassification of eligibility indicators for Medicaid MCO programs with inconsistent or missing enrollment labels eMethods 4. Linking CMS datasets to identify dental MCO enrollment eMethods 5. Derivation of adult dental enrollment by delivery system, 2016–2022 eTable. States without adult dental managed care organizations, 2016–2022 eFigure 1. Trends in state adoption of dental Medicaid managed care programs by delivery model and enrollment mandate, 2016–2022 eFigure 2. State level MCO dental benefit coverage areas and enrollment mandate for low-income adults, 2016-2022 eFigure 3. Distribution of Medicaid managed care program types offering any adult dental benefits, by service type, 2016–2022 eFigure 4. State-level Medicaid MCO dental benefit structure (carved-out vs carved-in), 2016-2022 eFigure 5. State-level dental benefit generosity by MCO and FFS, 2016-2022 eFigure 6. Trends in alignment of adult dental benefits between Medicaid MCO and FFS programs, 2016–2022 eFigure 7. Distribution of dental benefit coverage among adult Medicaid MCO enrollees, by program type, 2016–2022 eFigure 8. Number of dental Medicaid managed care program adult enrollment by offerings, by state, 2022 eReferences [file jamahealthforum-e256958-s001.pdf]

Supplemental Online Content

Elani HW, Zhao N, Wallace J, Sommers BD. Dental coverage through Medicaid managed care vs fee-for-service. *JAMA Health Forum*. Published online February 27, 2026.
doi:10.1001/jamahealthforum.2025.6958

Appendix	Description	Page
**eMethods 1**	Study sample construction and subgroup dentitions	2
**eMethods 2**	Identifying Dental MCO Program Offerings and Adult Eligibility, 2016–2022	4
**eMethods 3**	Reclassification of eligibility indicators for Medicaid MCO programs with inconsistent or missing enrollment labels.	5
**eMethods 4**	Linking CMS datasets to identify dental MCO enrollment	6
**eMethods 5**	Derivation of adult dental enrollment by delivery system, 2016–2022	7
**eTable**	States without adult dental Managed Care Organizations, 2016–2022	8
**eFigure 1**	Trends in state adoption of dental Medicaid managed care programs by delivery model and enrollment mandate, 2016–2022	9
**eFigure 2**	State level MCO dental benefit coverage areas and enrollment mandate for low-income adults, 2016-2022	10
**eFigure 3**	Distribution of Medicaid managed care program types offering any adult dental benefits, by service type, 2016–2022	11
**eFigure 4**	State-level Medicaid MCO dental benefit structure (carved-out vs carved-in), 2016-2022	12
**eFigure 5**	State-level dental benefit generosity by MCO and FFS, 2016-2022.	13
**eFigure 6**	Trends in alignment of adult dental benefits between Medicaid MCO and FFS programs, 2016–2022	14
**eFigure 7**	Distribution of dental benefit coverage among adult Medicaid MCO enrollees, by program type, 2016–2022	15
**eFigure 8**	Number of dental Medicaid managed care program adult enrollment by offerings, by state, 2022	16

This supplemental material has been provided by the authors to give readers additional information about their work.

© 2026 Elani HW et al. *JAMA Health Forum*.

eMethods 1. Study sample construction and subgroup dentitions

Program	Data sources
**1. Total MCO enrollment (unduplicated)**	
	a. Centers for Medicare & Medicaid Services (CMS) Medicaid Data - Managed Care Enrollment Summary. https://data.medicaid.gov/dataset/52ed908b-0cb8-5dd2-846d-99d4af12b369.
	b. Kaiser Family Foundation (KFF) - Medicaid Managed Care Market Tracker. https://www.kff.org/state-category/medicaid-chip/
**2. MCO enrollment by programs and plans**	
	CMS Medicaid Data - Managed Care Enrollment by Program and Plan. https://data.medicaid.gov/dataset/0bef7b8a-c663-5b14-9a46-0b5c2b86b0fe
**3. MCO offering characteristics, including dental information**	
	CMS Medicaid Data – Managed Care Programs by State (by different years). https://data.medicaid.gov/datasets?fulltext=Managed%20Care%20Programs%20by%20State
**4. Dental MCO enrollment by programs and plans**	
	Direct data is not available.
	Calculation: We derive these estimates by matching data from 2 and 3 (see eMethod 2 for details). For certain state-years, the dental information has been adjusted based on our own investigation.[1]
**5. Total dental MCO enrollment**	
	Direct data is not available.
	Calculation: Aggregated from 4
**6. Child MCO enrollment**	
	CMS - Early and Periodic Screening, Diagnostic and Treatment (EPSDT) Participation Report. https://www.medicaid.gov/medicaid/benefits/early-and-periodic-screening-diagnostic-and-treatment
**7. Child dental MCO enrollment in California**	
	The California Department of Health Care Services (DHCS). https://www.dhcs.ca.gov/services/Pages/DMCPerformanceMeasures.aspx
**8. Adult MCO enrollment**	
	Direct data is not available.
	Calculation: Estimated by subtracting 6 from 1 (using KFF data) [2]
**9. Adult dental MCO enrollment**	
	Direct data is not available.
	Calculation: Estimated by subtracting 6 (7 for California) from 5 [3]
**10. Adult MCO enrollment without any dental services**	
	Direct data is not available.
	Calculation: Estimated by subtracting 6 and 9 from 1 (selectively from CMS or KFF) [4]
**11. Adult Medicaid enrollment**	
	CMS Medicaid Data - T-MSIS-based monthly Medicaid and CHIP enrollment counts by eligibility group. [5] https://data.medicaid.gov/dataset/ea9b7db3-db71-4663-b4e1-67e11d1d4fcc
**12. Adults FFS enrollment**	
	Direct data is not available.
	Calculation: Estimated by subtracting 9 from 11[6]
**13. States FFS dental Indicators**	
	Elani, Hawazin; Benjamin D. Sommers; Ichiro Kawachi; Renuka Tipirneni; Meredith Rosenthal, 2025, "Database of State-Level Variations in Medicaid Adult Dental Coverage", https://doi.org/10.7910/DVN/LPHSLV, Harvard Dataverse.
**14. Adult dental FFS enrollment**	
	Direct data is not available.
	Calculation: Calculated by multiplying 12 by 13.

© 2026 Elani HW et al. *JAMA Health Forum*.

Notes.

1. In Arkansas, during 2021–2022, dental services were carved out and provided through a dental PAHP. In Indiana, in 2016, the Hoosier Healthwise program, non-dental MCO, did include dental benefits. [1]

2. Estimation discrepancies may arise from differences in data collection methods across sources. Total MCO enrollment figures reported by CMS and KFF represent point-in-time counts, while child enrollment data derived from EPSDT records reflect the number of beneficiaries ever enrolled for at least 90 continuous days during the federal fiscal year. This discrepancy may lead to negative adult enrollment estimates in certain state-year with few adult MCO enrollees or during transitions from MCO to FFS, such as Georgia (2016), Missouri (2019), Maine (2022), and Wyoming (2021, 2022). In these cases, we imputed a value of zero to represent the absence of adult MCO enrollment.

3. In certain state-years, such as Iowa (prior to 2021), Michigan (2016–2022), and North Dakota (2016–2022), Medicaid dental MCO programs were exclusively available to adults. In these instances, subtraction of child enrollment from total MCO enrollment was not necessary, as the programs did not serve pediatric populations.

4. In certain states and years, particularly where dental services were carved out and delivered through dental PAHPs, total MCO enrollment figures differed between the CMS and KFF data sources. For this analysis, we used CMS-reported enrollment data for states operating dental PAHPs and KFF-reported data for states without PAHPs, to better align with the structure of dental benefit delivery.

5. To approximate annual enrollment, we used June enrollment counts as representative of each year, consistent with the point-in-time methodology used by both CMS and KFF, which report totals as of July 1. However, for Rhode Island, we made exceptions: we used May 2018 and January 2020 enrollment figures to represent 2018 and 2019, respectively. This adjustment was necessary due to an unexplained and abrupt decline in reported enrollment between June 2018 and December 2019. Notably, the T-MSIS data quality assessment flagged Rhode Island's 2018 and 2019 Medicaid enrollment data as having "High concern," further justifying the use of alternative time points for those years. [2]

6. Similar to Note 2, discrepancies in data collection and measurement methods across datasets or years may result in minor estimation errors. In particular, Hawaii (2022), Michigan (2019 and 2022), and New Jersey (2022) exhibited small negative estimates, with absolute errors of less than 2% of total adult Medicaid enrollment. In In these instances, we assigned a value of zero for adult MCO enrollment. However, in Vermont (2020–2022), the absolute estimation errors exceeded 5%, and as a result, these data were excluded from the analysis. [2]

© 2026 Elani HW et al. *JAMA Health Forum*.

eMethods 2. Identifying dental MCO program offerings and adult eligibility, 2016–2022

We identified all Medicaid Managed Care Organization (MCO) programs from the CMS annual "Managed Care Programs by State" dataset.[3] Programs classified under the Program of All-Inclusive Care for the Elderly (PACE) were excluded due to their restrictive eligibility criteria and minimal enrollment. Numerous PACE programs were active between 2016 and 2022, and most included dental services. Specifically, 30 PACE programs offered dental services in 2022, 29 in 2021, 30 in 2020, 30 in 2019, 29 in 2018, 28 in 2017, and 29 in 2016. However, they accounted for less than 0.03% of total dental MCO enrollment and were therefore not included in the analysis.

To determine the eligible population for each MCO program, we used the following indicators:

- Adult indicators: "Low-income adults covered under ACA Section," "Low-income adults not covered under ACA Section," and "Individuals receiving Limited Benefits".
- Child indicators: "Non-Disabled Children," "Children with Special Health Care Needs," and "Foster Care and Adoption Assistance Children".
- Hybrid indicator: "Aged, Blind or Disabled Children or Adults".

We categorized programs into two groups: serving adults or serving children only. Specifically, a program was classified as serving adults if any of the adult indicators were labeled as "Mandatory," "Voluntary," "Both," or "Varies." If none of the adult indicators applied but the program was marked as serving "Aged, Blind or Disabled Children or Adults", we conducted a manual review to determine the target population. For example, although the following programs were indicated as serving "Aged, Blind or Disabled Children or Adults", they were determined to be children only: "Rady Children's Hospital San Diego," "Healthy Kids Dental," "Children's Medicaid Dental Services," "Rite Smiles Dental Program," "STAR Kids," and all the dental PAHP programs in Utah.

Using the above approach, 11 programs across select states and years were either incorrectly classified or could not be classified due to missing or ambiguous data. To address these cases, we modified the indicator labels using information from other years and manual review, as detailed in eMethods 3. From 2016 to 2022, we identified 462 dental MCO program-year records, 387 of them served adults, while 75 were classified as child-only.

© 2026 Elani HW et al. *JAMA Health Forum*.

eMethods 3. Reclassification of eligibility indicators for Medicaid MCO programs with inconsistent or missing enrollment labels

State	Year	Program	Indicator	Original label	Modified label	Label in other years
**District of Columbia**	2016	Medicaid Managed Care Program	Low-income adults covered under ACA Section	Unknown	Mandatory	Mandatory: 2017-2022
**Georgia**	2016	Georgia Families 360o	Foster Care and Adoption Assistance Children	Unknown	Mandatory	Mandatory: 2017-2022
**Missouri**	2016	Mo Healthnet Managed Care/1915b	Low-income adults not covered under ACA Section	Unknown	Mandatory	Mandatory: 2017-2022
**Mississippi**	2016	Mississippi Coordinated Access Network	Low-income adults not covered under ACA Section	Unknown	Mandatory	Mandatory: 2017-2022
**North Dakota**	2021, 2022	North Dakota Medicaid Expansion	Low-income adults covered under ACA Section	Unknown	Mandatory	Mandatory: 2016-2020
**New Jersey**	2021	NJ FamilyCare	Low-income adults covered under ACA Section	Unknown	Mandatory	Mandatory: 2017-2020, 2022
**New York**	2016	Health and Recovery Plans	Low-income adults not covered under ACA Section	Unknown	Voluntary	Voluntary: 2017-2022
**Oklahoma**	2016	SoonerCare Choice	Low-income adults not covered under ACA Section	Unknown	Mandatory	Mandatory: 2017-2022
**Texas**	2016	STAR	Low-income adults not covered under ACA Section	Unknown	Mandatory	Mandatory: 2017-2022
**Texas**	2016	STAR Kids	Foster Care and Adoption Assistance Children	Unknown	Mandatory	Mandatory: 2017-2022
**Wisconsin**	2016	Care4Kids	Foster Care and Adoption Assistance Children	Unknown	Voluntary	Voluntary: 2017-2022

Note. Original label is from the Centers for Medicare & Medicaid Services (CMS) annual "Managed Care Programs by State" dataset, 2016–2022.

© 2026 Elani HW et al. *JAMA Health Forum*.

eMethods 4. Linking CMS datasets to identify dental MCO enrollment, 2016–2022

Dental services can be delivered through various types of MCO programs, including comprehensive MCOs, dental PAHPs, PCCMs, and others. Except dental PAHPs, for all other MCO types, dental benefits are only partially offered, depending on specific programs and plans. As a result, we do not have direct enrollment counts for adults receiving dental services through these programs. To estimate adult dental MCO enrollment, we constructed a linked dataset by merging two CMS sources: “Managed Care Enrollment by Program and Plan”[4] and “Managed Care Programs by State”.[3] The resulting dataset includes both enrollment and dental benefit information for all Medicaid MCO programs and plans across all states from 2016 to 2022.

To extract dental benefit details for each plan listed in the enrollment dataset, we identified corresponding records in the program characteristics dataset using a two-step matching strategy. First, we conducted an exact match on year, state, and program type to generate a set of candidate plans. Next, we applied a combination of fuzzy string matching and cosine similarity metric over the Term Frequency–Inverse Document Frequency (TF-IDF) to align “program name + plan name” and identify the optimal match within each candidate set.[5] This approach yielded high matching accuracy, correctly linking 99.5% of plans, which together accounted for 99.3% of total MCO enrollment over the study period.

Two-step matching strategy for CMS datasets linkage

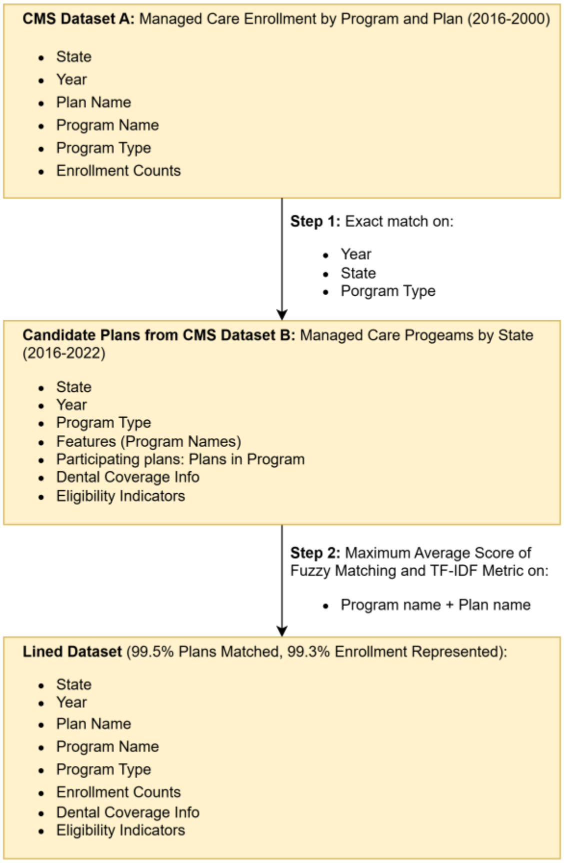

© 2026 Elani HW et al. *JAMA Health Forum*.

eMethods 5. Derivation of adult dental enrollment by delivery system, 2016–2022

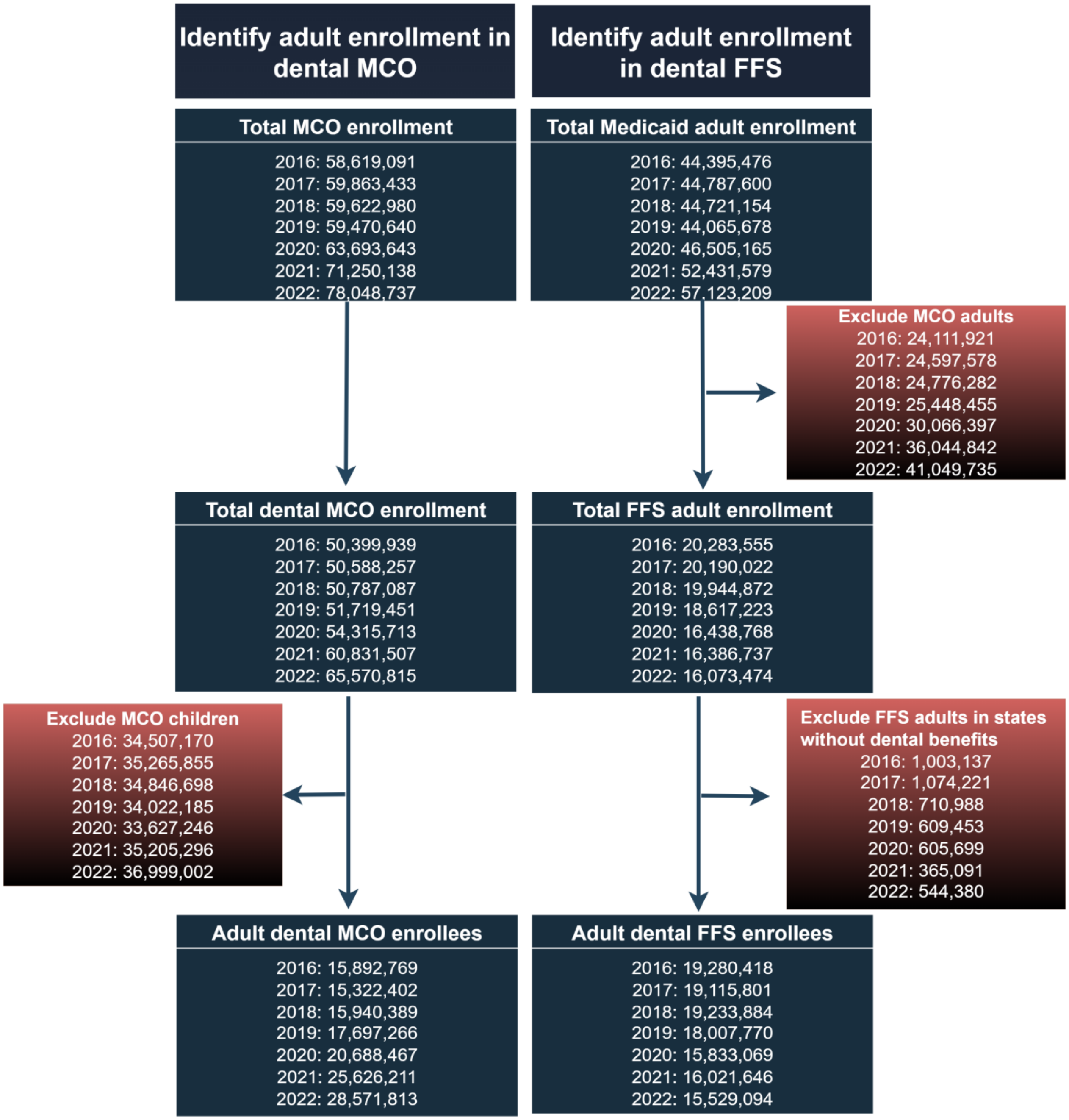

© 2026 Elani HW et al. *JAMA Health Forum*.

eTable. States without adult dental Managed Care Organizations, 2016–2022

Year	Number of states	States without any dental MCO for adults
2016	12	Alabama, Arkansas, Delaware, Maryland, Maine, North Carolina, Nebraska, New Hampshire, South Carolina, Utah, Virginia, Wyoming.
2017	14	Alabama, Arkansas, Colorado, Delaware, Maryland, Maine, North Carolina, New Hampshire, Nevada, Oklahoma, South Carolina, South Dakota, Virginia, Wyoming.
2018	12	Alabama, Delaware, Illinois, Maryland, Maine, Montana, North Carolina, New Hampshire, South Carolina, South Dakota, Utah, Wyoming.
2019	11	Delaware, Maryland, Maine, Montana, North Carolina, New Hampshire, Oklahoma, South Carolina, South Dakota, Utah, Wyoming.
2020	13	Alabama, Colorado, Delaware, Maryland, Maine, Montana, North Carolina, New Hampshire, Oklahoma, South Carolina, South Dakota, Utah, Wyoming.
2021	11	Alabama, Maryland, Maine, Montana, North Carolina, New Hampshire, Oklahoma, South Carolina, South Dakota, Utah, Wyoming.
2022	11	Alabama, Maryland, Maine, Montana, North Carolina, New Hampshire, South Carolina, Rhode Island, South Dakota, Utah, Wyoming.

Note. This table lists states that did not offer any adult dental benefits through Medicaid MCOs in each year of the study period. The number of states without dental MCOs varied annually. Alaska and Connecticut did not operate any Medicaid managed care programs during the 2016–2022 period.

© 2026 Elani HW et al. *JAMA Health Forum*.

eFigure 1. Trends in state adoption of dental Medicaid managed care programs by delivery model and enrollment mandate, 2016–2022

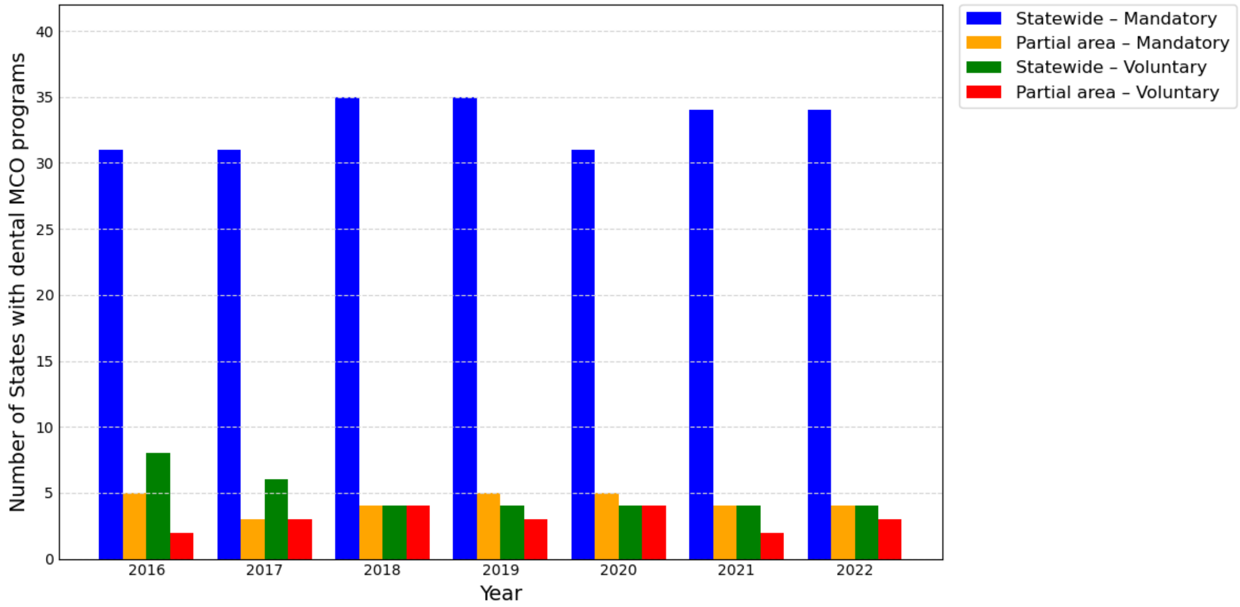

Note. Authors' analysis of data from the Centers for Medicare & Medicaid Services Medicaid Datasets, 2016–2022. Programs are classified as statewide (covering the entire state) or partial-area (limited to specific regions). Enrollment may be mandatory (required for beneficiaries) or voluntary (beneficiaries may opt out).

© 2026 Elani HW et al. *JAMA Health Forum*.

eFigure 2. State level MCO dental benefit coverage areas and enrollment mandate for low-income adults, 2016-2022

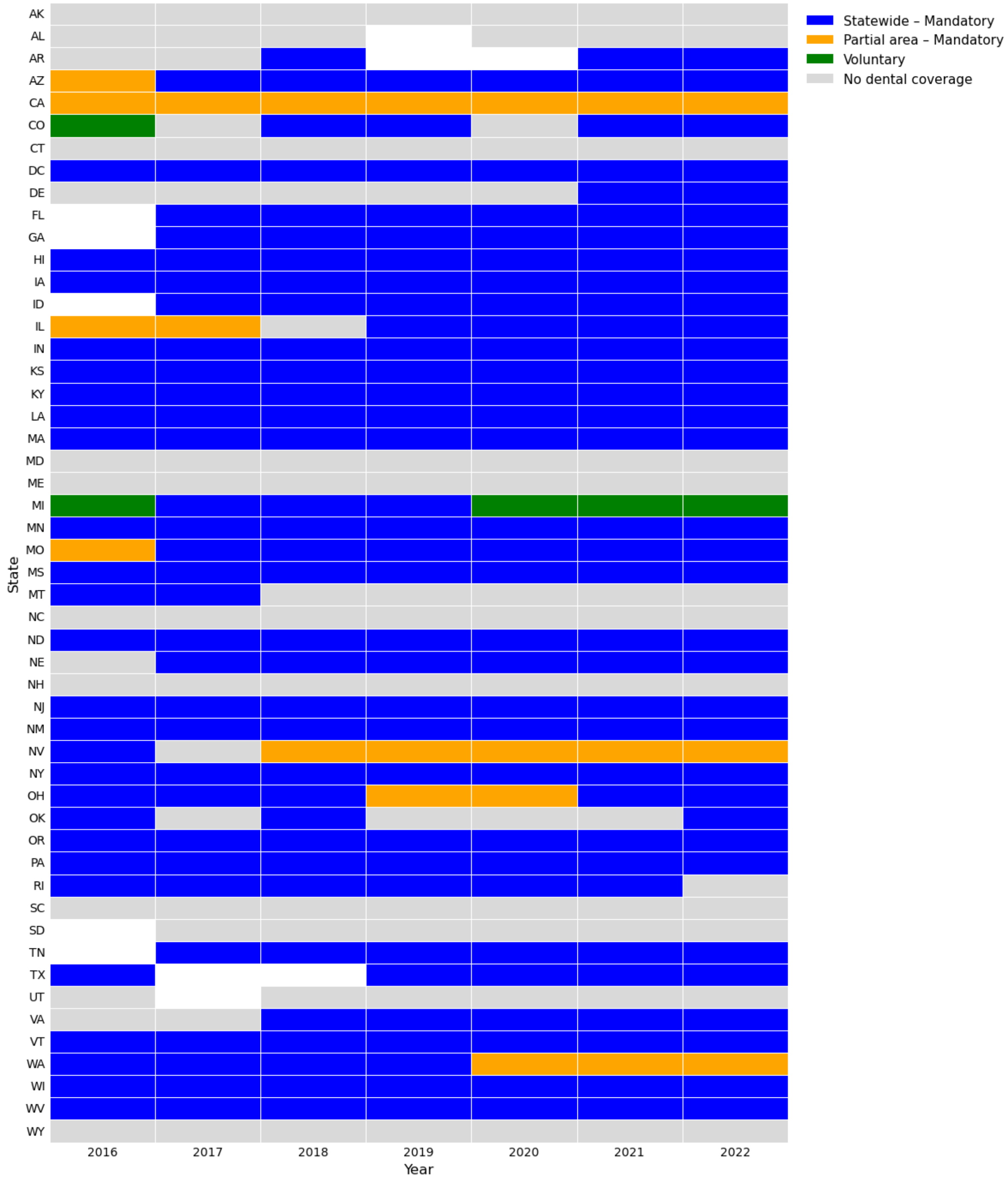

Note. Authors' analysis of data from the Centers for Medicare & Medicaid Services Medicaid Datasets for years 2016–2022. For states operated multiple MCO programs that provided dental benefits, we indicate the state-level status by selecting the highest-level program (we rank "State - Mandatory" > "Partial area - Mandatory" > "Voluntary") for low-income adults, regardless of ACA expansion status. White cells denote missing information for the corresponding states-years.

© 2026 Elani HW et al. *JAMA Health Forum*.

eFigure 3. Distribution of Medicaid managed care program types offering any adult dental benefits, by service type, 2016–2022

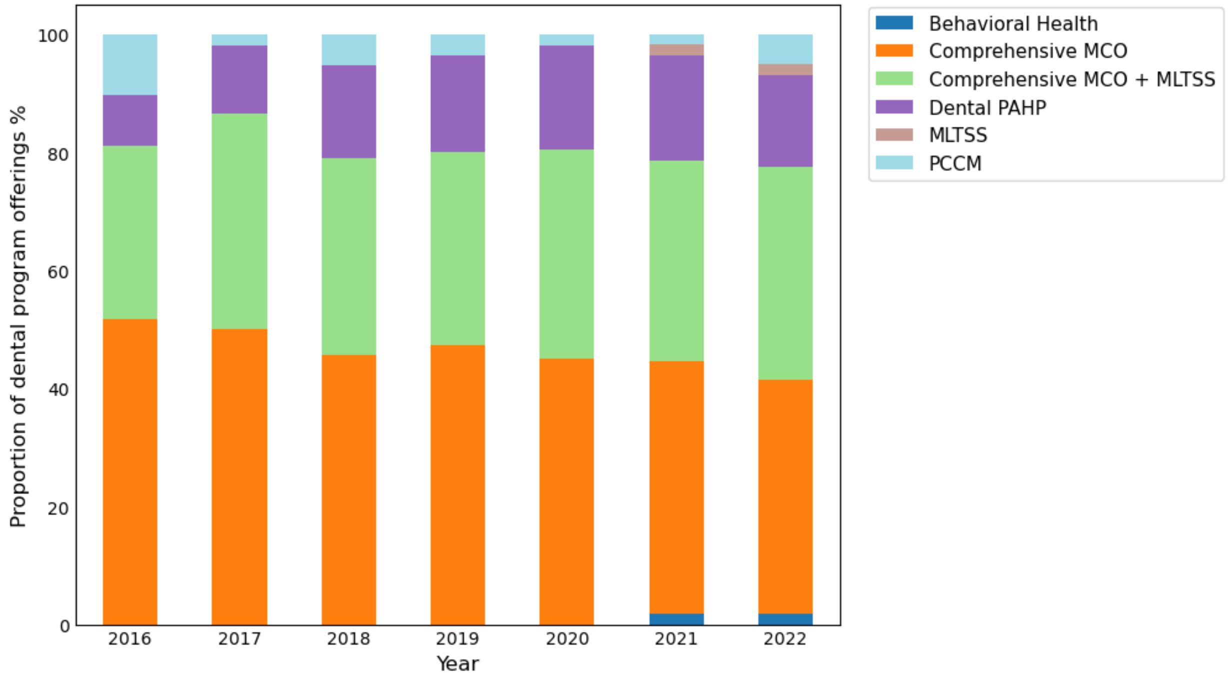

Note. Authors' analysis of data from the Centers for Medicare & Medicaid Services Medicaid Datasets for years 2016–2022. Counts are duplicated, meaning a single state may operate multiple program types concurrently. Program types include Comprehensive MCOs (medical and dental), Comprehensive MCOs with MLTSS (Managed Long-Term Services and Supports), Dental PAHPs (standalone dental Prepaid Ambulatory Health Plans), Behavioral Health, PCCM (Primary Care Case Management), and MLTSS-only programs.

© 2026 Elani HW et al. *JAMA Health Forum*.

eFigure 4. State-level Medicaid MCO dental benefit structure (carved-out vs carved-in), 2016-2022

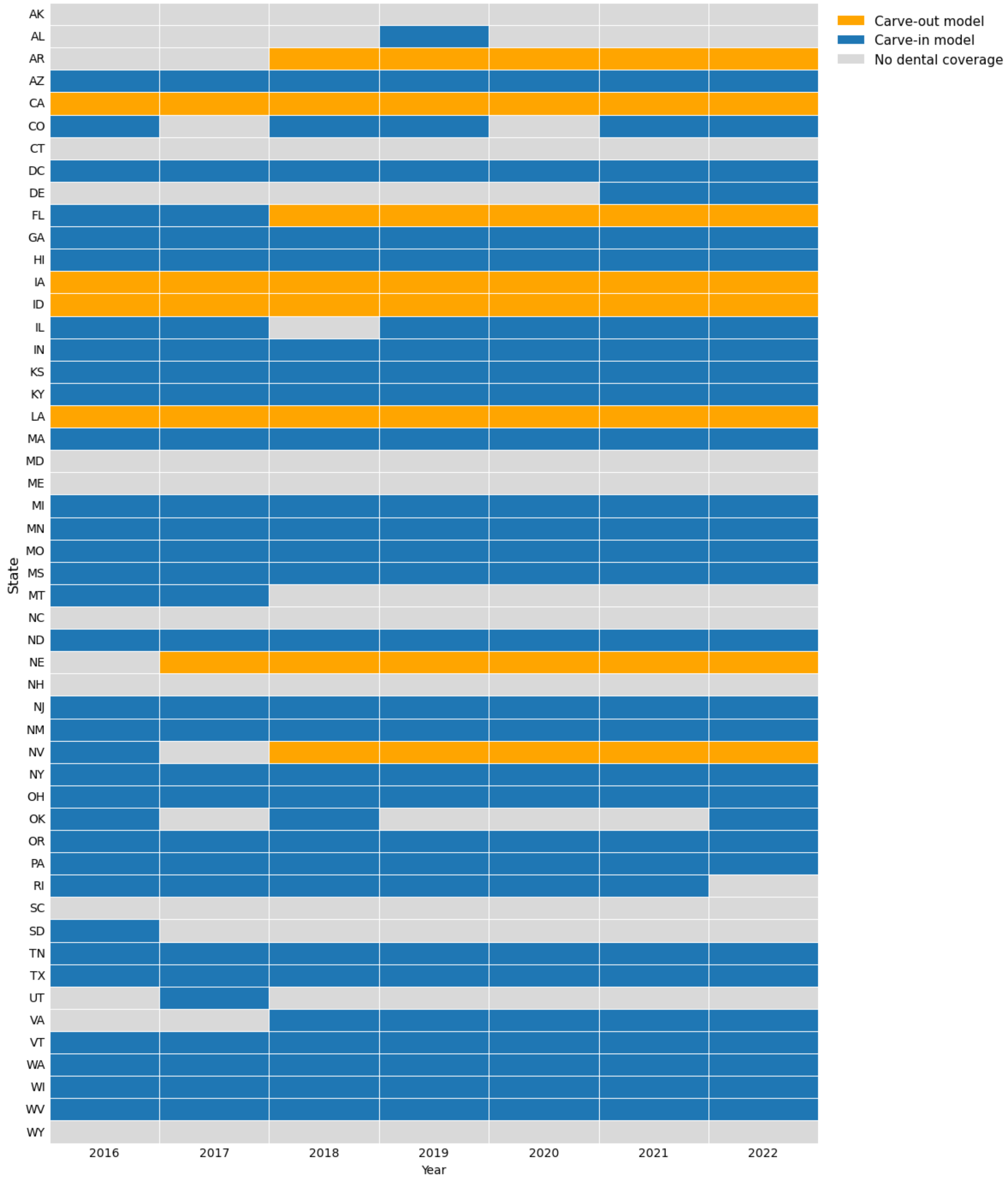

Note. This heatmap displays annual state-level MCO dental benefit structures, distinguishing between carved-out and carved-in delivery models. Authors' analysis of data from the Centers for Medicare & Medicaid Services Medicaid Datasets for years 2016–2022.

© 2026 Elani HW et al. *JAMA Health Forum*.

eFigure 5. State-level dental benefit generosity by MCO and FFS, 2016-2022

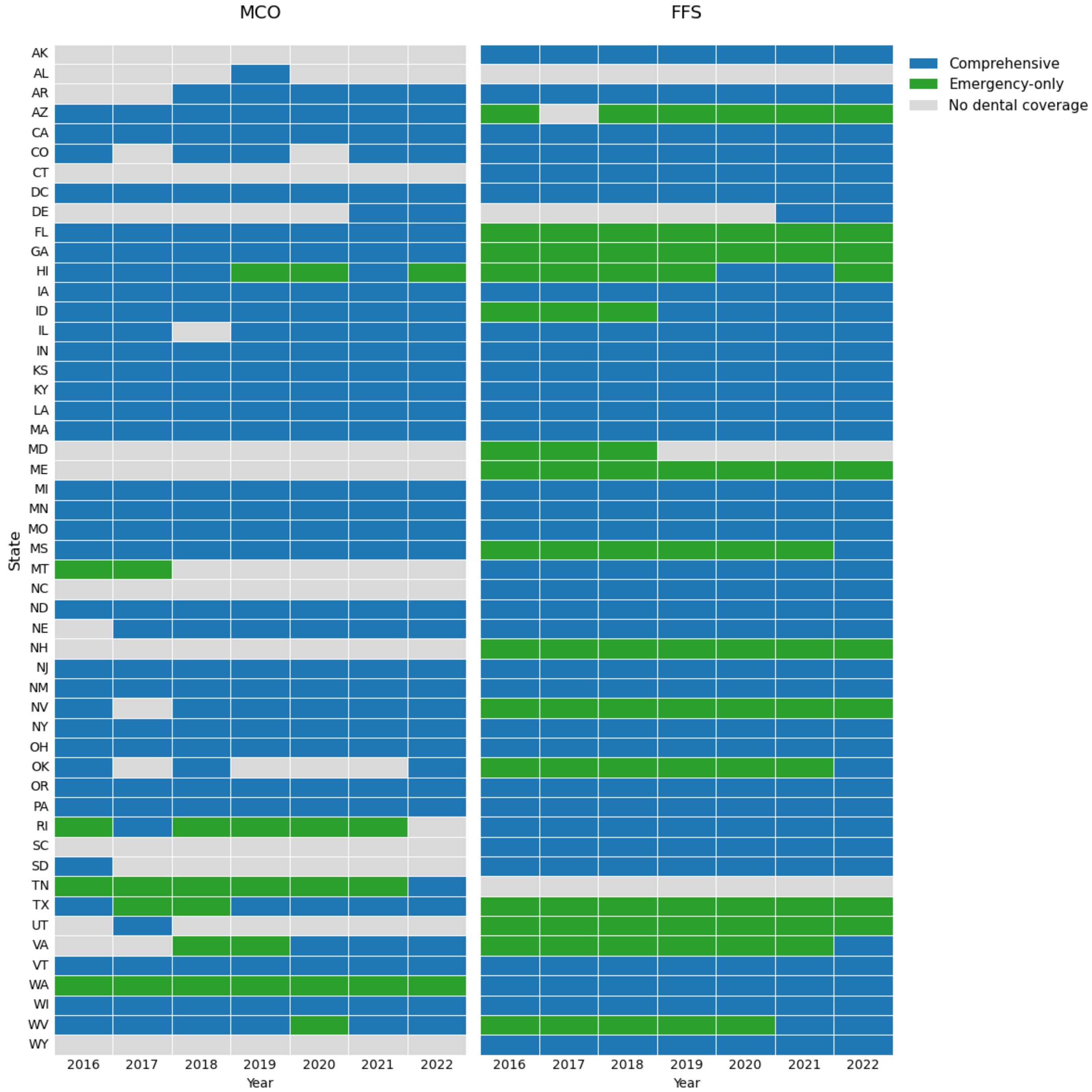

Note. This heatmap compares the annual state-level dental benefit generosity between MCO and FFS programs. Authors' analysis of data from the Centers for Medicare & Medicaid Services Medicaid Datasets for years 2016–2022.

© 2026 Elani HW et al. *JAMA Health Forum*.

eFigure 6. Trends in alignment of adult dental benefits between Medicaid MCO and FFS programs, 2016–2022

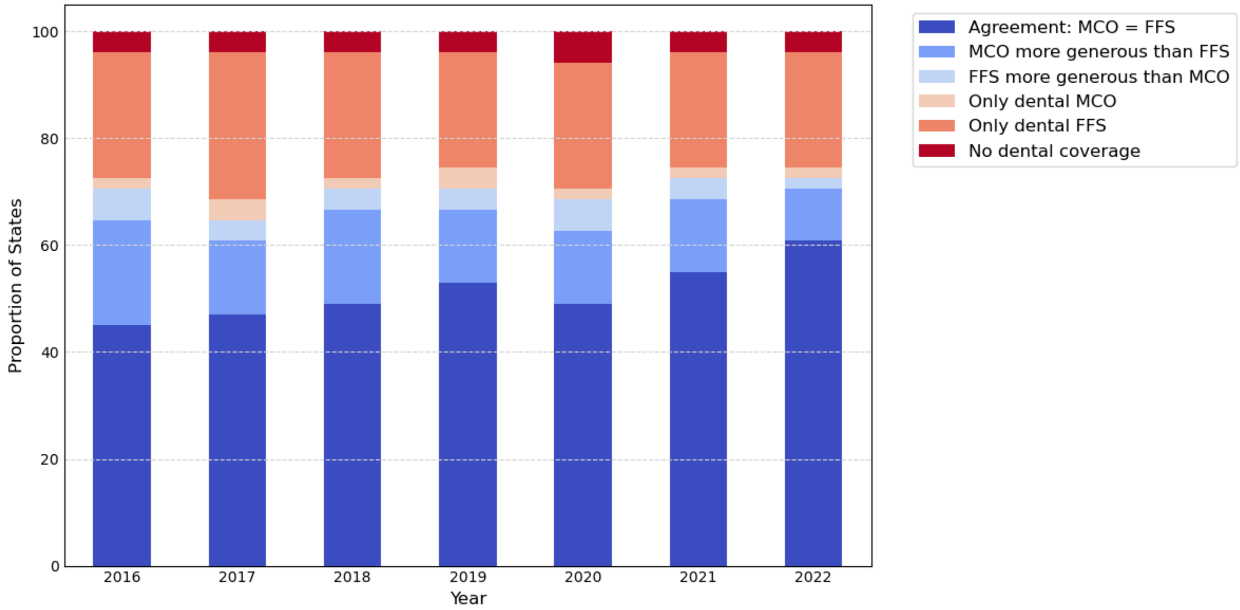

Note. Authors' analysis of data from the Centers for Medicare & Medicaid Services Medicaid Datasets for years 2016–2022. "Agreement: MCO = FFS" indicates states where MCOs and FFS provided the same level of dental benefits in a given year. "MCO more generous than FFS" and "FFS more generous than MCO" indicate states where both delivery models offered adult dental benefits but differed in benefit generosity. "Only dental MCO" indicates states that offered adult dental benefits exclusively through MCO programs, whereas "Only dental FFS" indicates states that provided adult dental benefits only through FFS Medicaid. "No dental coverage" reflects states with no adult dental benefits in either Medicaid delivery model.

© 2026 Elani HW et al. *JAMA Health Forum*.

eFigure 7. Distribution of dental benefit coverage among adult Medicaid MCO enrollees, by program type, 2016–2022

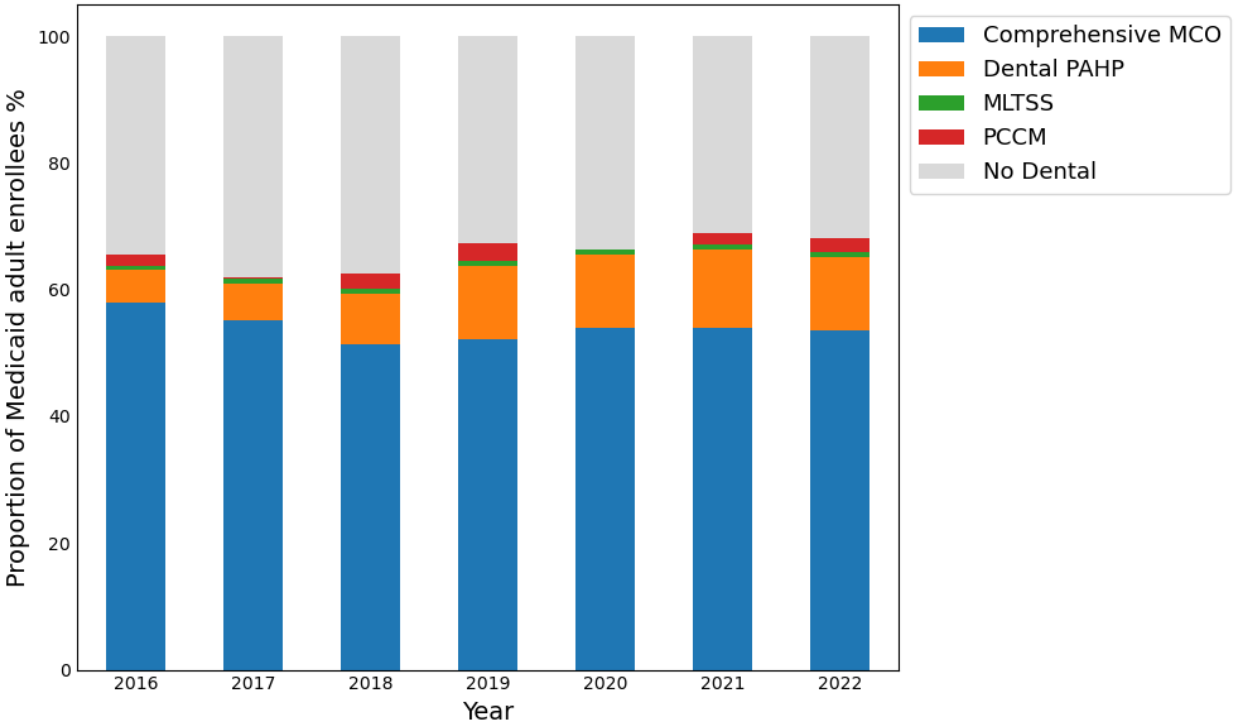

Note. Authors' analysis of data from the Centers for Medicare & Medicaid Services Medicaid Datasets for years 2016–2022. Comprehensive MCOs: Managed care plans covering both medical and dental services. Dental PAHPs: Prepaid Ambulatory Health Plans that offer standalone dental coverage. MLTSS: Managed Long-Term Services and Supports programs that may include dental benefits. PCCM: Primary Care Case Management programs with dental components. "No dental" refers to adults enrolled in MCO programs that did not include any dental benefit in a given year.

© 2026 Elani HW et al. *JAMA Health Forum*.

eFigure 8. Number of dental Medicaid managed care program adult enrollment by offerings, by state, 2022

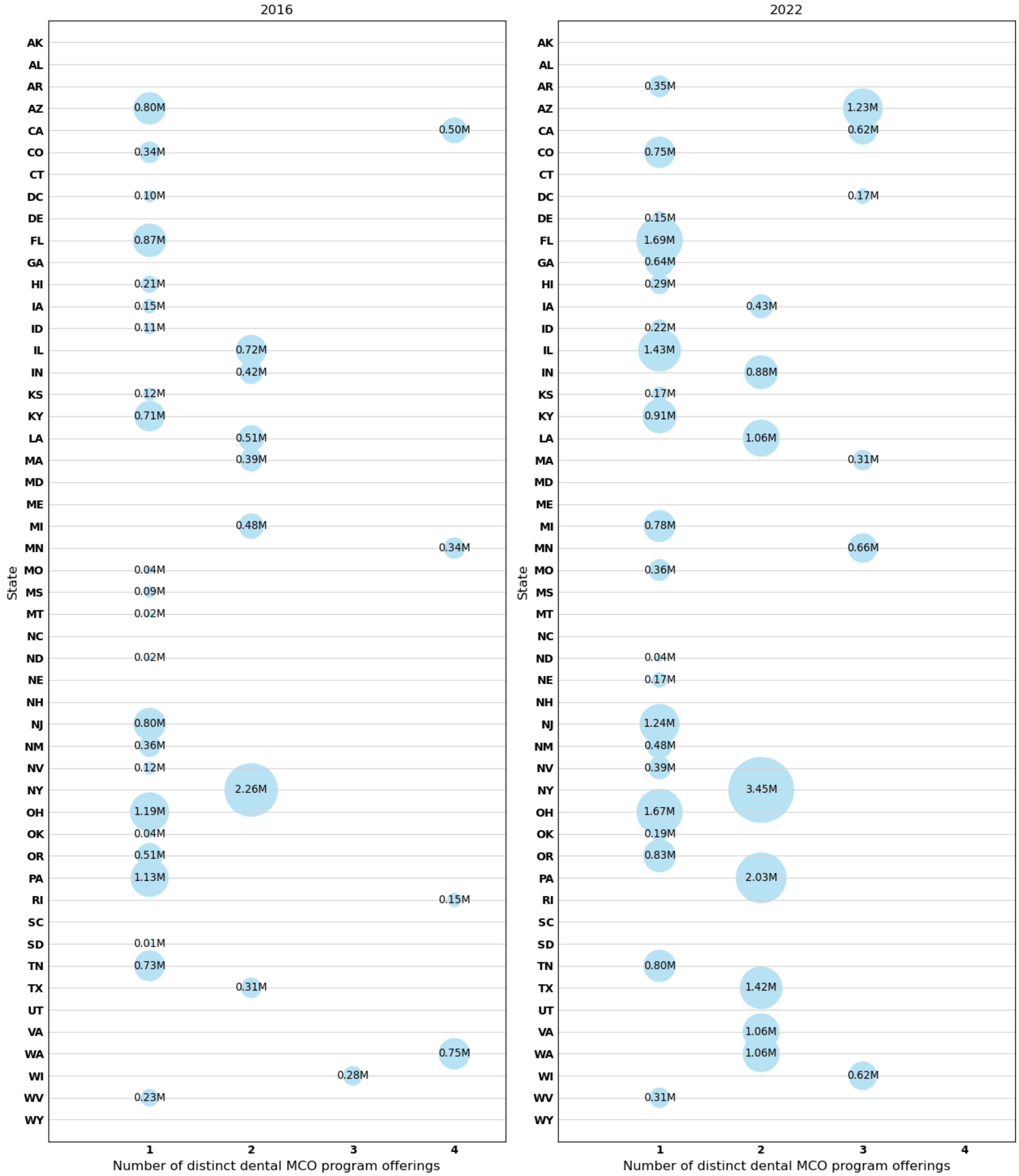

Note. Each bubble represents a U.S. state in 2016 and 2022. The horizontal axis shows the number of distinct Medicaid dental managed care program offerings (e.g., comprehensive MCOs, Dental PAHPs) operating in the state. The size of each bubble reflects the total number of adult Medicaid enrollees covered under these programs, labeled in millions (M).

© 2026 Elani HW et al. *JAMA Health Forum*.

eReferences

1. Indiana Family and Social Services Administration, Office of Medicaid Policy and Planning. *Indiana Medicaid-Managed Care Quality Strategy Plan 2016*. FSSA. Published 2016. https://www.in.gov/fssa/ompp/files/2016-Indiana-Medicaid-Quality-Strategy-Plan.pdf.

2. Centers for Medicare & Medicaid Services. *Medicaid Data Quality Atlas: Data Quality information by topic*. Medicaid.gov. https://www.medicaid.gov/dq-atlas/landing/topics/info.

3. Centers for Medicare & Medicaid Services. *Managed Care Programs by State*. Data.Medicaid.gov. https://data.medicaid.gov/datasets?fulltext=Managed%20Care%20Programs%20by%20State.

4. Centers for Medicare & Medicaid Services. *Managed Care Enrollment by Program and Plan.* Data.Medicaid.gov. https://data.medicaid.gov/dataset/0bef7b8a-c663-5b14-9a46-0b5c2b86b0fe.

5. Alodadi M, Janeja VP. Similarity in patient support forums using TF-IDF and cosine similarity metrics. In: *2015 International Conference on Healthcare Informatics (ICHI)*. IEEE; 2015:521–522. https://doi.org/10.1109/ICHI.2015.99.

© 2026 Elani HW et al. *JAMA Health Forum*.
