# Supplementary material for: Dental Coverage Through Medicaid Managed Care vs Fee-for-Service
Source: JAMA Health Forum. 2026 Feb 27;7(2):e256958. doi: 10.1001/jamahealthforum.2025.6958 (PMC12949447; doi:10.1001/jamahealthforum.2025.6958)
Supplement: Supplement 2. — Data sharing statement [file jamahealthforum-e256958-s002.pdf]

# Data Sharing Statement

Elani. Dental Coverage Through Medicaid Managed Care vs Fee-For-Service. *JAMA Health Forum*. Published February 27, 2026. doi:10.1001/jamahealthforum.2025.6958

**Data**
**Data available:** No
